# Buriti Oil Emulsions as Affected by Soy Protein Isolate/High-Methoxyl Pectin Ratio, Oil Content and Homogenization Pressure

**DOI:** 10.17113/ftb.58.02.20.6210

**Published:** 2020-06

**Authors:** Mírian Luisa Faria Freitas, Ana Paula Badan Ribeiro, Vânia Regina Nicoletti

**Affiliations:** 1 School of Engineering (FAEN), Federal University of Grande Dourados (UFGD), Dourados-Itahum Road Km 12, Cidade Universitária, Dourados, Mato Grosso do Sul, 79.804-970, Brazil; 2 Department of Food Technology (DTA), School of Food Engineering (FEA), University of Campinas (UNICAMP), Bertrand Russel Street, Cidade Universitária, Campinas, São Paulo, 13.083-970, Brazil; 3 São Paulo State University (UNESP), Institute of Biosciences, Humanities and Exact Sciences (IBILCE), Campus São José do Rio Preto, 2265 Cristóvão Colombo Street, Jardim Nazareth, São José do Rio Preto, São Paulo, 15.054-000, Brazil

**Keywords:** buriti oil, dispersed systems, electrostatic interaction, high-methoxyl pectin, soy protein isolate, emulsion rheology

## Abstract

**Research background:**

Emulsion technology is a suitable way of encapsulating, protecting and releasing hydrophobic bioactive compounds for application in food industries, but they are thermodynamically unstable systems. Good results have been achieved for emulsions stabilized by protein-polysaccharide complexes subjected to high-pressure homogenization. Improved stabilization of oil-in-water emulsions results from electrostatic complexes formed between proteins and polysaccharides at pH lower than the protein isoelectric point, which adsorb at the oil-water interface. In addition, polysaccharides contribute to emulsion stability by increasing viscosity of the continuous phase. The aim of this work is to investigate the production of carotenoid-rich buriti oil emulsions using soy protein isolate and high-methoxyl pectin as stabilizers.

**Experimental approach:**

Using a rotatable central composite experimental design, we assessed the effects of oil content, soy protein isolate/high-methoxyl pectin ratio and homogenization pressure on the stability, droplet size, electrical conductivity, electrical charge, microstructure and rheological behaviour of the emulsions.

**Results and conclusions:**

An optimized emulsion was produced with 28% buriti oil, 55% soy protein isolate, and homogenization pressure of 380·10^5^ Pa. This emulsion was stable for at least seven days, presenting reduced average droplet size, low electrical conductivity and high modulus of negative charges. The mechanical spectra showed that the emulsion behaved as a viscoelastic gel under oscillatory, non-destructive shearing, whereas shear-thinning behaviour took place under steady shear conditions.

**Novelty and scientific contribution:**

The optimized buriti oil emulsions stabilized by soy protein isolate and high-methoxyl pectin could be suitable for fat substitution, energy reduction and carotenoid enrichment in food products, such as dairy and bakery products, ice cream, salad sauces and vegetable-based cream.

## INTRODUCTION

Emulsion technology is suitable for encapsulation, protection and modulation of the release of hydrophobic bioactive compounds for use in food and pharmaceutical industries. However, emulsions are thermodynamically unstable systems and require addition of emulsifiers and/or stabilizers, as well as the use of energy (homogenization process) to attain kinetic stability (*1*). Proteins are surface-active molecules that act as surfactants and prevent coalescence of dispersed droplets: they spontaneously migrate from the bulk phase to the oil-water interface, as free energy of proteins at the interface is lower than in the continuous phase (*2*). The emulsion stabilizing properties of proteins may be enhanced by their potential electrostatic interactions with charged polysaccharides, which results in complex amphiphilic structures. These complexes or association colloids do not require a special legislation and may improve emulsion stability and textural attributes (*3*-*6*).

Soy protein isolate (SPI) and high-methoxyl pectin (HMP) are examples of a protein and anionic polysaccharide that may form electrostatic complexes when pH is lower than the protein isoelectric point (*5*). Soy protein isolate contains at least 90% protein, being practically free of lipids and carbohydrates. The main components of these proteins are β-conglycinin (7S) and glycinin (11S) fractions, which represent more than 85% of the total soybean proteins (*7*). These two fractions have emulsifying activity, being able to stabilize emulsions by forming an adsorbed layer at the oil/water interface (*8*). Pectin, one of the most complex natural macromolecules, is the most commonly used stabilizer in protein-based acidic beverages, and contributes positively to flavour, stability and texture of final products, even when added in small amounts (*9*, *10*). High-methoxyl pectins possess more than half of their carboxyl groups in the methyl ester form and at low pH the highly hydrated and charged carboxylate groups turn to uncharged and slightly hydrated carboxylic acid groups, leading to association of the pectin molecules and formation of a polymer network that entraps water (*11*). The technological characteristics of SPI/HMP complexes as emulsion stabilizers, which are natural ingredients that comply with vegetarian and vegan consumer demands, make these ingredients very interesting choices to obtain a fine dispersion of components in food.

At pH lower than 4.6, which corresponds to the isoelectric point of SPI, the negatively charged HMP interacts with the protein positive charges to form a biopolymer double layer, preventing droplet coalescence and stabilizing the emulsion (*12*, *13*). Protein-polysaccharide complexes enhance emulsion stability through electrostatic and/or steric phenomena, by modifying interfacial rheological properties and increasing emulsion viscosity (*14*). In order to provide steric stabilization, it is desirable that, in addition to the hydrophobic groups that will remain permanently attached to the oil droplet surface, the macromolecular structure at the oil-water interface has a large fraction of hydrophilic chain segments that will protrude from the surface and increase the stabilizing layer thickness. At the same time, if this macromolecular structure contains charged groups that contribute to increasing the net repulsive electrostatic interactions, it would help to prevent aggregation of adjacent droplets caused by the attractive van der Waals forces (*4*).

In addition to using surface-active agents, like emulsifiers, to reduce surface tension of dispersed systems, achievement of kinetic stability of oil-in-water (O/W) emulsions requires the use of energy to reduce droplet sizes and thus prevent creaming, which is a consequence of the difference in density between disperse and continuous phases. The homogenization step is also of crucial importance to modulate physicochemical and organoleptic properties, such as texture, taste, appearance and stability of emulsified systems (*15*). High-pressure homogenization equipment is effective in reducing droplet sizes of the pre-existing coarse emulsions: the fluid is pumped through a small adjustable orifice, located between the valve and its support, generating a combination of intense shear and cavitation in turbulent regime, thus promoting droplet size reduction (*16*). Good results have been reported for emulsions stabilized with protein-polysaccharide complexes subjected to high-pressure homogenization (*6*).

Knowledge of the rheological properties of emulsions is important for several reasons, which include the design of processing equipment (*17*), estimation of sensory attributes related to texture, and flow properties. In addition, rheological assays can provide fundamental information on structural organization and interactions between emulsion components (*15*). The rheological behaviour of concentrated emulsions ranges between the limit of dilute emulsions – with a linear dependence of viscosity on droplet concentration and negligible interactions between droplets, and emulsions containing closely packed spherical droplets – in which it is impossible to increase the number of droplets without deforming the existing ones. The increase of droplet concentration in emulsions results in increased Newtonian viscosity at low shear rates, followed by appearance of strong non-Newtonian effects. As the droplet concentration increases, the Newtonian viscous flow is replaced by a viscoplastic behaviour with a step-like decrease of the apparent viscosity that is typical for multi-component systems with a coagulate structure formed by the dispersed phase, and reflects the breakage of the structure at a certain shear rate that is associated with a yield stress. The increase in the concentration also enhances the influence of the droplet size on the emulsion rheology, as it influences the volume-to-surface area ratio. In addition, other rheological effects may appear at high concentrations, such as shearing time effects. These time effects are due to structural arrangements between the interfacial layers in the closely packed droplets, which are destroyed by deformation and restored at rest. The interaction between droplets and evolution of their shape in the flow can also result in viscoelastic effects (*2*, *15*, *18*, *19*).

Oil-in-water emulsions have been recognized as appropriate systems to encapsulate and vehiculate hydrophobic nutrients and functional compounds, such as carotenoid-rich vegetable oil. Palm trees belonging to the *Aracaceae* family that includes buriti (*Mauritia flexuosa*) are among the most useful vegetable resources in the Amazon region. Buriti oil has a high concentration of monounsaturated fatty acids, with values higher than those found in olive oil or Brazil nuts, foods known to have high nutritional quality oil due to the properties of reducing blood cholesterol (*20*). Regardless of this valuable functional properties, buriti palm tree is not yet commercially cultivated and processed, so that buriti oil consumption is relatively low, being used mostly as a specialty food ingredient and in the cosmetics industry. A major interest has arisen because buriti oil contains β-carotene in higher concentrations than foods widely consumed by Brazilians, such as guava, pitanga, papaya, passion fruit, carrots and other fruits from the Amazon region, including palm nuts, peach palm and tucumã (*21*, *22*). Additionally, buriti oil contains high levels of tocopherols, making it a reliable source of antioxidant compounds (*23*, *24*). When used as food additives, carotenoids and tocopherols are relatively unstable because they are sensitive to light, oxygen, temperature and self-oxidation (non-enzymatic cleavage), which causes their rapid degradation (*25*, *26*). Emulsification and microencapsulation may be effective for protecting carotenoids and tocopherols from adverse effects of environment during storage (*1*).

Considering this context, the aim of this work is to investigate the production of buriti oil emulsions by high pressure homogenization using soy protein isolate and high-methoxyl pectin as stabilizers, as well as characterizing the resulting systems by evaluating their stability, droplet size, electrical conductivity, electrical charge, rheological behaviour and morphology. Emulsions containing the carotenoid-rich buriti oil stabilized by SPI/HMP could be valuable structured systems applied in foods such as dairy and bakery products, ice cream, salad sauces and vegetable-based cream for fat substitution, energy reduction and carotenoid enrichment.

## MATERIAL AND METHODS

### Materials

The raw materials used were buriti oil (Amazon Oil Industry^TM^, Ananindeua, Pará, Brazil), soy protein isolate (SPI) (Tovani Benzaquen Ingredientes^TM^, São Paulo, São Paulo, Brazil) and high-methoxyl pectin (HMP) (CP Kelco^TM^, Matão, São Paulo Brazil). Preparation of samples required deionized water, 1 M solutions of sodium azide, hydrochloric acid or sodium hydroxide (all Dinâmica^TM^, Indaiatuba, São Paulo, Brazil) and McIlvaine buffer (pH=3.5) prepared using disodium phosphate and citric acid (Dinâmica^TM^).

### Preparation of buriti oil emulsions

Oil-in-water emulsions were prepared with different mass fractions of oil (10-30%) and SPI in the continuous phase (50-80%), and different applied homogenization pressures (200·10^5^-400·10^5^ Pa). These were the three independent variables. The HMP content was not included as an independent variable in the experimental design since it was added in the amount necessary to attain a fixed total mass fractions of biopolymers (SPI+HMP) in the continuous phase of the emulsion.

All the emulsions were prepared using McIlvaine buffer at pH=3.5, which is below the isoelectric point of SPI and allows the biopolymers in the solution to acquire opposite charges in a sufficient amount to form electrostatic complexes that remain dispersed in the aqueous phase (*13*). Stock solutions of SPI (3 g/100 g) were prepared by dispersing the protein in half of the required water and adjusting pH=11.0±0.05 with NaOH for complete solubilization, then subjecting them to magnetic stirring for 3 h, leaving to rest overnight (25 °C) for complete hydration, and finally completing the water content with buffer pH=3.5. Stock solutions of HMP (2 g/100 g) were prepared by dispersion in buffer solution at pH=3.5, then 3 h under magnetic stirring, and overnight hydration at 25 °C. Sodium azide 0.04% was added to stock solutions to prevent microorganism growth (*13*).

Emulsions were produced by following the method described by Kaltsa *et al.* (*27*), with some modifications. Buriti oil was dispersed in the SPI stock solution using Ultra Turrax stirrer (T-25; IKA, Staufen, Baden-Württemberg, Germany) at 15 000 rpm for 4 min, followed by the addition of HMP stock solution, and dispersion at 15 000 rpm for 4 min using the same stirrer. After this procedure, the coarse emulsion was homogenized at high pressure in a two-stage homogeniser (Lab Series APV-2000; SPX Flow Technology, Charlotte, NC, USA), without sample recirculation.

### Experimental design

The assays were carried out according to a 2^3^ rotatable central composite design (RCCD) to analyze the effects of three independent variables (*28*). Five assays were added at the central point of the experimental design, totalizing 19 assays (Table 1). The following polynomial model was fitted to the data:


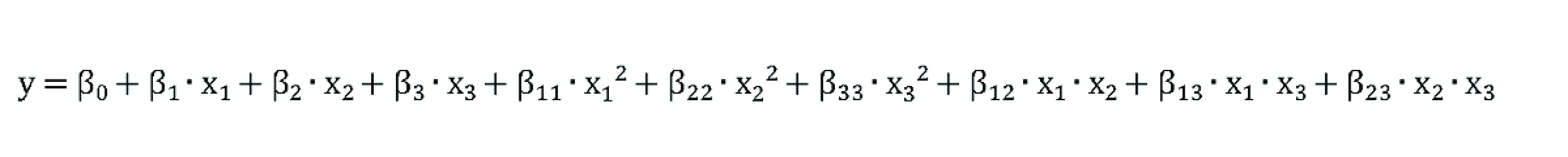


**Table 1:** Buriti oil emulsion assays with coded variables and real values according to 2^3^ rotatable central composite design (RCCD) and final emulsion composition

Assay	RCCD					Emulsion composition			
*w*(oil)/% (x_1_)		*w*(SPI)/% (x_2_)		*p*/10^5^Pa (x_3_)			*w*/%	
					Oil	SPI	HMP	water
O14S56P240	-1 (14)	-1 (56)		-1 (240)		14.00	1.20	0.93	83.87
O14S56P360	-1 (14)	-1 (56)		+1 (360)		14.00	1.20	0.93	83.87
O14S74P240	-1 (14)	+1 (74)		-1 (240)		14.00	1.70	0.60	83.70
O14S74P360	-1 (14)	+1 (74)		+1 (360)		14.00	1.70	0.60	83.70
O26S56P240	+1 (26)	-1 (56)		-1 (240)		26.00	1.02	0.80	72.18
O26S56P360	+1 (26)	-1 (56)		+1 (360)		26.00	1.02	0.80	72.18
O26S74P240	+1 (26)	+1 (74)		-1 (240)		26.00	1.45	0.50	72.05
O26S74P360	+1 (26)	+1 (74)		+1 (360)		26.00	1.45	0.50	72.05
O10S65P300	-1.68 (10)	0 (65)		0 (300)		10.00	1.49	0.81	87.70
O30S65P300	+1.68 (30)	0 (65)		0 (300)		30.00	1.16	0.63	68.21
O20S50P300	0 (20)	-1.68 (50)		0 (300)		20.00	0.96	0.96	78.08
O20S80P300	0 (20)	+1.68 (80)		0 (300)		20.00	1.74	0.44	77.82
O20S65P200	0 (20)	0 (65)		-1.68 (200)		20.00	1.30	0.70	78.00
O20S65P400	0 (20)	0 (65)		+1.68 (400)		20.00	1.30	0.70	78.00
O20S65P300	0 (20)	0 (65)		0 (300)		20.00	1.30	0.70	78.00
O20S65P300	0 (20)	0 (65)		0 (300)		20.00	1.30	0.70	78.00
O20S65P300	0 (20)	0 (65)		0 (300)		20.00	1.30	0.70	78.00
O20S65P300	0 (20)	0 (65)		0 (300)		20.00	1.30	0.70	78.00
O20S65P300	0 (20)	0 (65)		0 (300)		20.00	1.30	0.70	78.00

*w*(SPI)^a^=(*m*_SPI_/(*m*_SPI_+*m*_HMP_))∙100. SPI=soy protein isolate, HMP=high-methoxyl pectin

where y is the response variable, x_1_ is the oil mass fraction (*m*(oil)/*m*(emulsion))/%, x_2_ is the SPI mass fraction (*m*(SPI)/*m*(SPI+HMP))/%, *x*_3_ is the homogenization pressure (Pa), and β_i_ and β_ij_ are the fitted coefficients.

The results were subjected to analysis of variance (ANOVA) and the fitted model was tested regarding the lack of fit and coefficient of regression using the software STATISTICA v. 7.0 (*29*). For all fitted models, the coefficient of regression was significant, whereas the lack of fit was not significant, suggesting good fit and accuracy of the models.

### Characterization of buriti oil emulsions

The emulsions were stored at 25 °C for a 7-day period after production. Then they were analyzed in triplicate regarding stability, droplet size, electrical conductivity, electrical charge and morphology using optical microscopy and scanning electron microscopy (SEM) and rheological behaviour. Confocal laser scanning microscopy (CLSM) was performed on the same day of emulsion preparation with fluorescent dyes to avoid fluorescence losses. For samples that show phase separation after 7 days, the analyses were performed using the samples from the creamed phase. The responses for the experimental RCCD were stability, droplet size, electrical conductivity and electrical charge. The analytical procedures are described as follows.

#### Stability

Transparent tubes containing the emulsions were closed and stored at 25 °C. When phase separation took place, a lower density upper phase (cream) was formed. The creaming index (CI), indicative of emulsion stability, was determined as the ratio of the lower phase height (*h*) and the emulsion initial height (*h*_0_) in the tube according to the following equation (*6*):


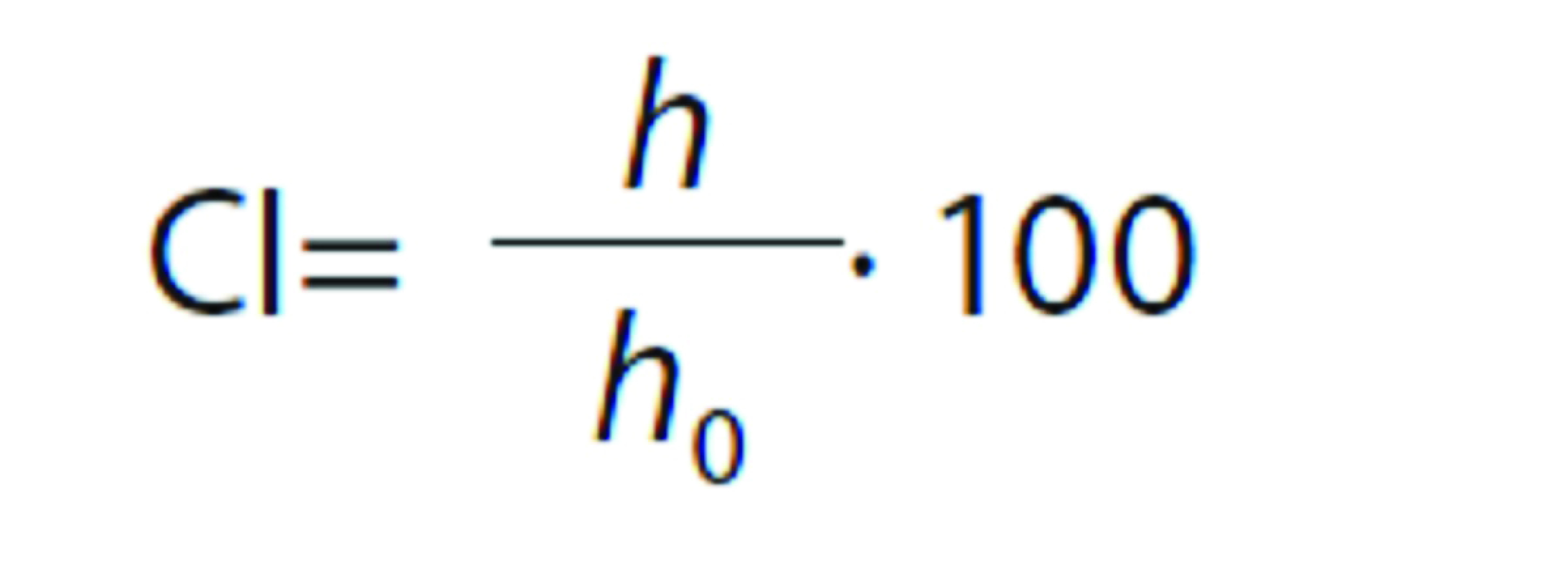


#### Droplet size by optical microscopy and laser diffraction

The average droplet size was evaluated using an optical microscope (OM model CX31; Olympus, Tokyo, Japan) with a 40× magnification objective coupled with a digital camera (SC30; Olympus) and software Image-Pro Plus 6.0 (*30*). A minimum number of 300 droplets to be measured to give significant results was defined after comparing the average sizes calculated from 100 to 800 droplets applying a Tukey's test. There were no significant differences (p>0.05) among the calculated average sizes, with satisfactory results for size distribution and standard deviation, when measuring 300 droplets or more.

In addition to optical microscopy, droplet size distribution was evaluated by laser diffraction (LD; Mastersizer 2000 with Hydro 2000S dispersion unit; Malvern, Malvern, Worcestershire, UK). The samples were dispersed in deionized water at 10% obscuration and kept under stirring during analysis. The refractive indices used for droplets and dispersant phase were 1.46 (buriti oil) and 1.33 (deionized water), respectively. Considering that some samples showed bimodal particle size distribution, the mode of the cumulative frequency distribution was adopted as the most representative particle size (*2*).

#### Electrical conductivity and charge analysis

The emulsion electrical conductivity was measured using a conductivity meter (Seven Compact S230-USP/EP; Mettler Toledo, Columbus, OH, USA) at room temperature (*31*).

Electrical charges were analyzed by zeta potential measurements (Zetasizer NanoZ; Malvern). The emulsion or cream phase samples (0.05%) were dispersed in ultrapure water followed by pH=3.5 adjustment using HCl solution to maintain the dispersing medium as close as possible to the emulsion continuous phase, according to the proposed method (*32*).

#### Rheological behaviour

Rheological analyses were performed on an AR-2000EX (TA Instruments, New Castle, DE, USA) rheometer with geometry of serrated parallel plates of 40 mm diameter and gap of 300 μm at 25 °C. To avoid droplet crushing, the distance between parallel plates (gap) was defined as being at least 10 times larger than the maximum droplet size (*33*). Viscosity curves were obtained from steady shear assays and the viscoelastic properties were determined from oscillatory shear tests.

#### Steady shear

Steady shear tests were performed following downward (100 to 0.1 s^-1^) and upward (0.1 to 100 s^-1^) shear rate ramps to obtain apparent viscosity *versus* shear rate curves. The Sisko viscosity model was fitted to the experimental data:


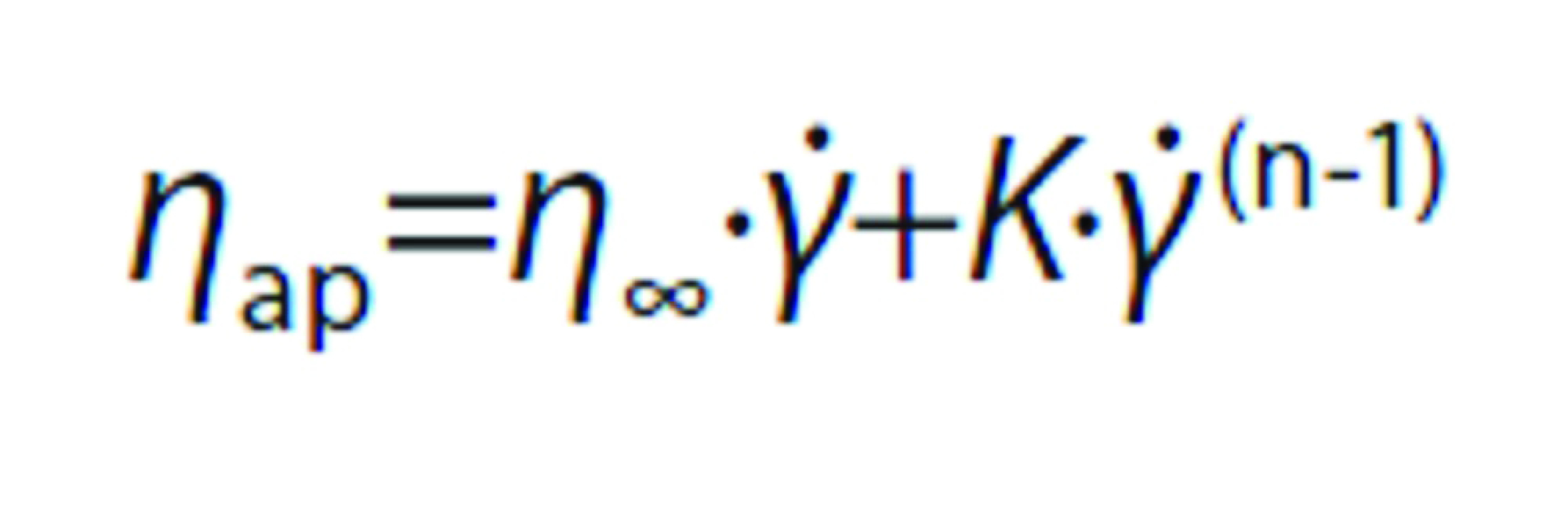


Where *η*_ap_ is the apparent viscosity (Pa·s), *η*_∞_ is the infinite-shear viscosity (Pa·s), *γ̇ *is the shear rate (s^-1^), is the consistency index (Pa·s^n^), and is the flow behaviour index (dimensionless) (*34*).

The Sisko model is interesting because it presents, in addition to the consistency index (*Κ*) and flow behaviour index (*n*), the infinite shear viscosity (*η*_∞_) of the samples. The infinite shear viscosity provides information on how the system behaves when exposed to high shear conditions, such as spraying or extrusion, which is relevant for food processing industries.

#### Oscillatory shear

The viscoelastic properties were evaluated along frequency sweeps between 0.03 and 30 rad/s at maximum deformation of 0.001, which was in the linear viscoelastic region. A power law model was applied to describe the frequency (*ω*) dependence on the storage (*G’*) and loss (*G”*) moduli, according to the following equations:


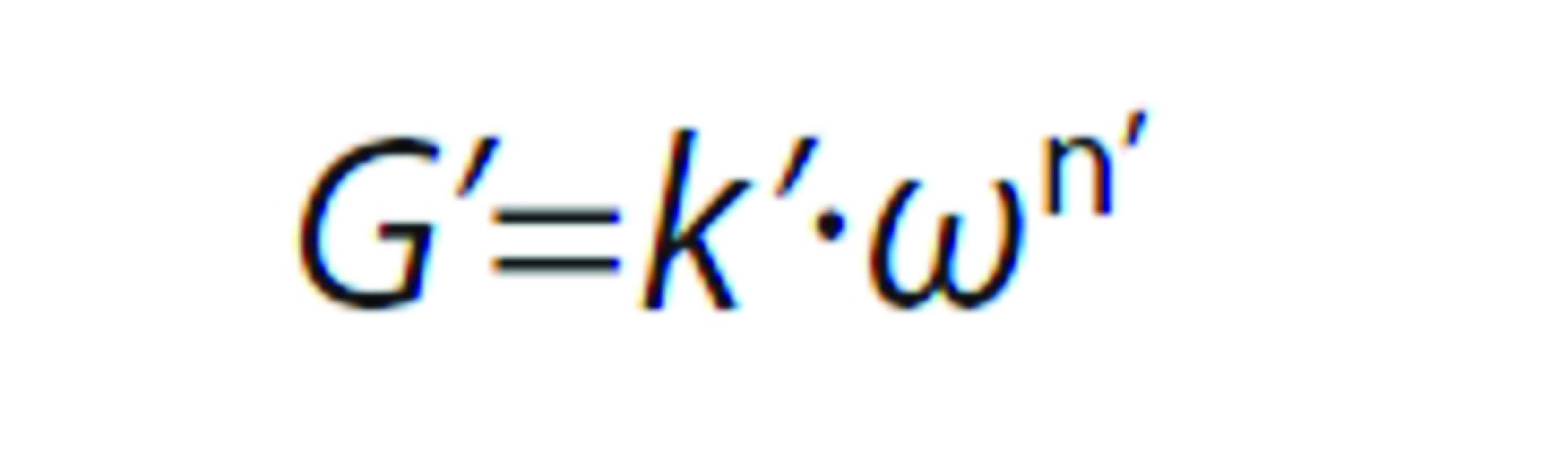


and


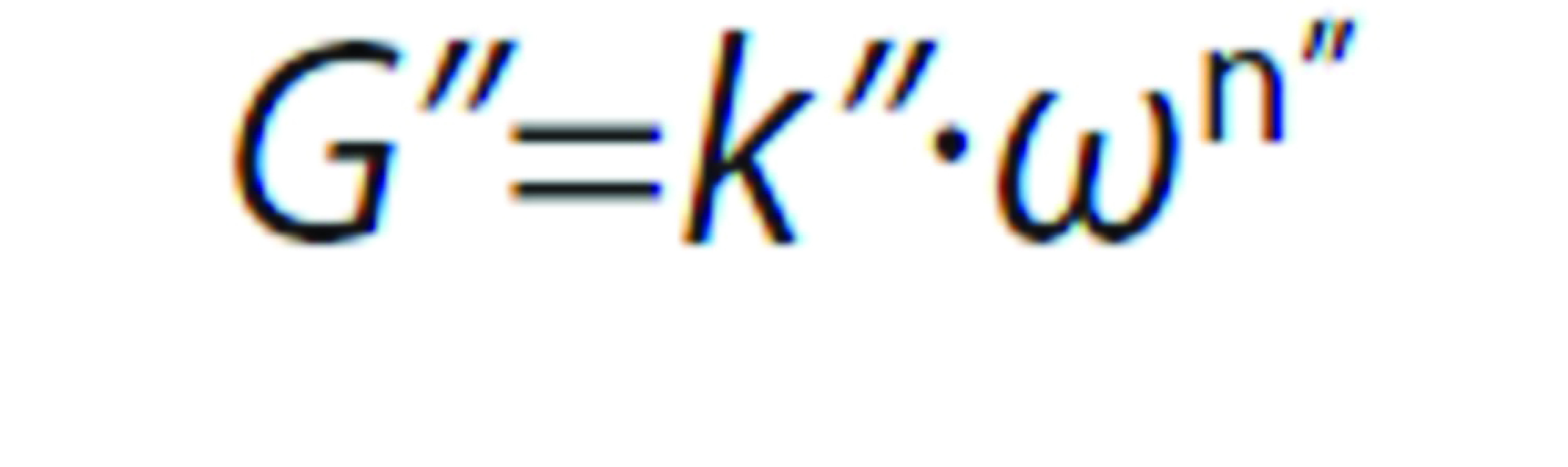


where *k’*, *n’, k”* and *n”* are the corresponding fitting parameters for *G’ vs*. *ω* and *G” vs*. *ω*, respectively. Values of *n’* and *n”* can provide useful information regarding the viscoelastic nature of materials. Results showing *n’*<*n”* and low magnitudes of *n’* and *n”* are typical of weak physical gels, especially at low frequency.

The fitting quality of Sisko (Eq. 3) and power law models (Eqs. 4 and 5) were evaluated by the coefficient of determination, R^2^, and the root mean squared errors, RMSE.

### Analyses of the emulsion morphology

#### Confocal laser scanning microscopy

Analyses by confocal laser scanning microscopy (CLSM) were carried out according to Lamprecht *et al.* (*35*) and Mukai-Correa *et al.* (*36*), using fluorescein isothiocyanate (FITC) to interact with the SPI, and Rhodamine B to interact with HMP. The dyes were dissolved in dimethyl sulfoxide (DMSO, 1 mg/mL) and 50 μL of dye per 2.5 g of polymer in stock solutions were added. The emulsions were then prepared as described in *Preparation of buriti oil emulsions*. Images were collected in a ZEISS LSM 710 microscope (ZEISS, Jena, Germany) with 40× magnification and the laser was adjusted to green/red fluorescence mode, at wavelengths of 488 and 543 nm to visualize FITC and Rhodamine B, respectively. Green and red fluorescent images were obtained from the two separate channels and, then, overlaid and analyzed by Zen 2010 software (*37*).

#### Scanning electron microscopy

Scanning electron microscopy (SEM) images were obtained in a TM 3000 (Hitachi, Tokyo, Japan) microscope with a 600× magnification. The samples were inserted into the chamber and immobilized by vacuum application during image capture.

### Experimental validation of the RCCD models

Experimental validation of the models resulting from the RCCD (Eq. 1) was carried out based on the results of stability, droplet size, electrical conductivity and electrical charge. Selected values for buriti oil and SPI mass fractions, and homogenization pressure were applied to produce an emulsion and the corresponding experimental results were compared with those predicted by the fitted models. In addition, this emulsion was characterized by rheological and morphological analyses (as described above).

## RESULTS AND DISCUSSION

The emulsions prepared with different buriti oil and SPI contents and subjected to various homogenization pressures, according to the RCCD in Table 1, were characterized regarding stability (CI), droplet size (by optical microscopy (OM) and laser diffraction (LD)), electrical conductivity, and electrical charge after 7 days of preparation. Treatments were coded with ten letters and numbers, for example: O14S74P360 meaning: 14% buriti oil, 74% SPI in wall material and homogenization pressure of 360·10^5^ Pa.

The obtained results (Table 2) were used to fit the empirical models (Eq. 1) and the regression coefficients for the coded second order polynomial fitted equations, as well as the determination coefficients (R*^2^*), for each one of the dependent variables given in Table 2. Some non-significant effects were eliminated, and the resulting equations were tested for adequacy and fitness by the analysis of variance (ANOVA). The fitted models were suitable, showing significant regression, low residual values, no lack of fit and satisfactory determination coefficients.

**Table 2:** Stability (creaming index, CI), droplet size (obtained by optical microscopy, OM, and laser diffraction, LD), electrical conductivity, and electrical charge (zeta potential) of emulsions and the coded second-order regression coefficients

Assay			CI/%		*d*(droplet OM)/μm			Span (droplet OM)		*d*(droplet LD)/μm			*σ*/(mS/cm)			*ζ*/mV	
O14S56P240			28.0±0.6		3.4			1.2		26.0±0.3			2.97±0.01			-20.3±1.3	
O14S56P360			11.7±0.6		2.9			1.1		38.9±0.6			3.08±0.01			-20.0±0.8	
O14S74P240			20.3±1.2		3.4			1.2		51.5±1.2			2.47±0.01			-16.3±1.2	
O14S74P360			22.3±1.6		2.4			1.3		51.7±1.9			2.81±0.01			-18.3±2.0	
O26S56P240			3.7±6.4		4.4			1.3		39.5±2.7			2.29±0.01			-20.5±1.0	
O26S56P360			0.0±0.0		4.5			1.9		42.4±0.6			2.18±0.01			-22.2±0.6	
O26S74P240			11.6±0.6		5.7			1.9		40.2±0.2			2.00±0.01			-15.2±1.3	
O26S74P360			14.7±1.6		4.7			1.3		35.0±0.5			2.38±0.01			-15.6±0.6	
O10S65P300			35.6±1.9		2.9			1.1		46.5±0.3			2.82±0.01			-20.4±0.8	
O30S65P300			0.0±0.0		6.8			2.4		40.6±0.8			2.16±0.01			-21.9±0.8	
O20S50P300			14.0±5.9		3.9			1.4		43.1±0.8			2.53±0.01			-22.3±0.7	
O20S80P300			16.4±1.4		4.3			1.7		66.3±0.8			2.02±0.01			-15.3±0.6	
O20S65P200			17.3±0.5		3.6			1.4		37.4±0.3			2.27±0.01			-19.6±1.2	
O20S65P400			10.1±1.5		2.9			0.9		53.1±1.2			2.47±0.01			-14.6±1.7	
O20S65P300			16.6±0.6		3.8			1.1		51.5±0.8			2.31±0.01			-20.7±1.8	
O20S65P300			14.5±1.5		3.9			1.4		50.3±1.5			2.70±0.01			-18.7±1.4	
O20S65P300			18.2±4.8		3.9			1.3		49.0±0.7			2.21±0.01			-17.8±1.4	
O20S65P300			14.0±0.9		3.4			1.3		48.0±0.4			2.38±0.01			-17.7±0.7	
O20S65P300			13.4±2.0		3.3			1.1		43.2±0.5			2.44±0.01			-20.6±0.4	
Regression analysis coefficient		CI/%				*d*(droplet OM)/μm	*d*(droplet LD)/μm				*σ*/(mS/cm)			*ζ*/mV		
	16.27			3.77					49.34			2.44			-19.45	
	-13.10			0.99					n.s.			-0.26			n.s.	
	7.66			n.s.					5.16			-0.12			2.16	
	-0.60			-0.25					2.73			0.09			n.s.	
	n.s.			0.37					-3.35			n.s.			n.s.	
	n.s.			n.s.					n.s.			n.s.			n.s.	
	n.s.			-0.19					-2.75			n.s.			0.87	
	9.28			0.26					-5.63			n.s.			n.s.	
	n.s.			n.s.					n.s.			n.s.			n.s.	
	10.66			-0.20					n.s.			n.s.			n.s.	
R^2^	0.88			0.94					0.71			0.74			0.66	

The values are mean with standard deviations; n.s.=non-significant (p>0.10)

### Buriti oil emulsion stability

The creaming index was significantly affected (p≤0.10) by the mass fractions of oil (β_1_) and SPI (β_2_), and homogenization pressure (β_3_). In addition, there were significant interactions between the oil and SPI mass fractions (β_12_), as well as between SPI mass fraction and homogenization pressure (β_23_) (Table 2). The oil content and homogenization pressure had negative effects, whereas SPI content had a positive effect on creaming index, being important to remember that the higher the CI, the higher the emulsion instability.

The increase of emulsion stability with increasing concentration of the dispersed phase is probably related to the higher viscosity associated with the increased concentration of emulsion droplets (*19*). In addition, the buriti oil used is a crude oil and has about 12% of diacylglycerols and monoacylglycerols, compounds that are recognized by their emulsifying properties (*24*). In such a way, it is possible to suggest that the increase in oil mass fraction leads to an increasing content of these compounds, which could contribute to lower CI values, or higher emulsion stability. On the other hand, lower SPI mass fractions in the continuous phase also imply higher HMP mass fractions (Table 1), which suggests that a minimum content of HMP is important to increase the continuous phase viscosity and prevent oil droplet diffusion, which could contribute to increase in the stability and lower CI values (*38*). In turn, higher homogenization pressures may contribute to the production of smaller oil droplets, resulting in a more homogeneous and, consequently, more stable system, with lower creaming index (*27*).

### Buriti oil emulsion droplet size

With respect to droplet size measured by OM, the variables with significant effects (p≤ 0.10) were the oil mass fraction (linear, β_1_, and quadratic, β_11_), homogenization pressure (β_3_ and β_33_), the interactions between the oil and SPI mass fractions (β_12_) and between the SPI mass fraction and homogenization pressure (β_23_). The oil mass fraction and the interaction between mass fractions of oil and SPI had positive effects on droplet size, while the homogenization pressure and the interaction between the SPI mass fractions and homogenization pressure had negative effects. This suggests that high HMP levels helped to stabilize the emulsions, although the smaller amount of SPI was not sufficient to produce emulsions with small droplets, since a larger amount of proteins would be needed to cover the larger interfacial area.

The droplet sizes determined by LD were represented by the distribution model and were significantly affected (p≤0.10) by the mass fractions of oil (β_11_) and SPI (β_2_), homogenization pressure (β_3_ and β_33_) and by the interaction between the oil and SPI mass fractions (β_12_). The SPI content and homogenization pressure had positive effects, whereas the oil content, homogenization pressure, and the interaction between the oil and SPI mass fractions had negative effects. It is interesting to note the large difference between the results for droplet size determined by OM and LD. Differences could also be observed in the variables that exerted significant effects on these results. The laser diffraction technique applied requires sample dilution, which does not happen in the optical microscopy technique. This fact led to the hypothesis that the dilution was not enough to completely disperse the oil droplets, which remained arranged in clusters. This hypothesis was confirmed by observing the emulsions at the same dilution in the optical microscope. Despite the observed differences, the results from LD are relevant, since this is the standard technique used for droplet size analysis in emulsions (*32*, *39*, *40*).

### Buriti oil emulsion electrical conductivity

The electrical conductivity was significantly affected (p≤0.10) by the mass fractions of oil (β_1_) and SPI (β_2_) and homogenization pressure (β_3_). The oil and SPI mass fractions had negative effects and the homogenization pressure had positive effect on the electrical conductivity of emulsions or on cream phases. These results can be explained considering that the systems with higher SPI mass fractions had lower HMP mass fraction and, that pectin helps to maintain oil droplets apart from each other. Therefore, in these systems there was a great possibility that oil droplets were flocculated, or even merged (coalescence) in the creamed phase, leading to lower electrical conductivity values. On the other hand, in emulsions subjected to higher homogenization pressures, smaller oil droplets and greater homogeneity are expected, thus, resulting in higher electrical conductivity values.

According to Bruttel (*41*), in oil-in-water emulsions, water has a high dielectric constant and is very polar, whereas the oil has a low dielectric constant and thus low polarity. When the water phase is bound to stabilizers, that is, the emulsion has maximum viscosity, its stability will also be maximum and, due to this fact, the electrical conductivity is minimal.

### Analysis of the buriti oil emulsion droplet charge

The zeta potential, or electrokinetic potential, is related to the electrophoretic mobility, gives a measure of the net charge on the surface of a macromolecule, and is used to describe the surface charge of polyelectrolytes of dispersed droplets. Like the electrical conductivity, zeta potential is related to emulsion stability. The more charged the droplets, the greater is the repulsion among them, which contributes to a more stable suspension. In order to prevent irreversible oil droplet flocculation, an emulsion should have a zeta potential greater than 25 mV (in modulus), characterizing a metastable system (*42*). All the emulsions prepared according to the RCCD had negative zeta potential, varying in the range of (-22.33±0.70) to (-14.60±1.74) mV (Table 2). At pH=3.5, SPI is positively charged and HMP has a slightly larger negative charge than SPI (*13*), thus suggesting that the presence of pectin was determinant for the net charge of emulsions, reinforcing the hypothesis that protein acts at oil/water interface and pectin, in addition to interacting with protein, covers droplets as a second layer, also acting as a stabilizer in the continuous phase (*4*).

Stable emulsions (O26S56P240, O26S56P360 and O30S65P300) showed high chargemodulus (Table 2). However, these values were lower than 25 mV and this may be an indication that these emulsions would not remain stable for a long time. The homogenization pressure might have contributed to this lower negative charge, since the smaller droplets resulted in larger surface area covered by SPI and available to interact with HMP. This greater interaction might contribute to further neutralization of available negative charges. In agreement with the above discussion, the variables with significant effects (p≤0.10) on zeta potential were SPI mass fraction (β_2_) and homogenization pressure (β_33_), both having positive effects on the zeta potential, *i.e.* increasing SPI mass fraction and homogenization pressure caused lower charge modulus.

### Rheological behaviour of buriti oil emulsions

#### Steady shear emulsion behaviour

**Fig. 1:**
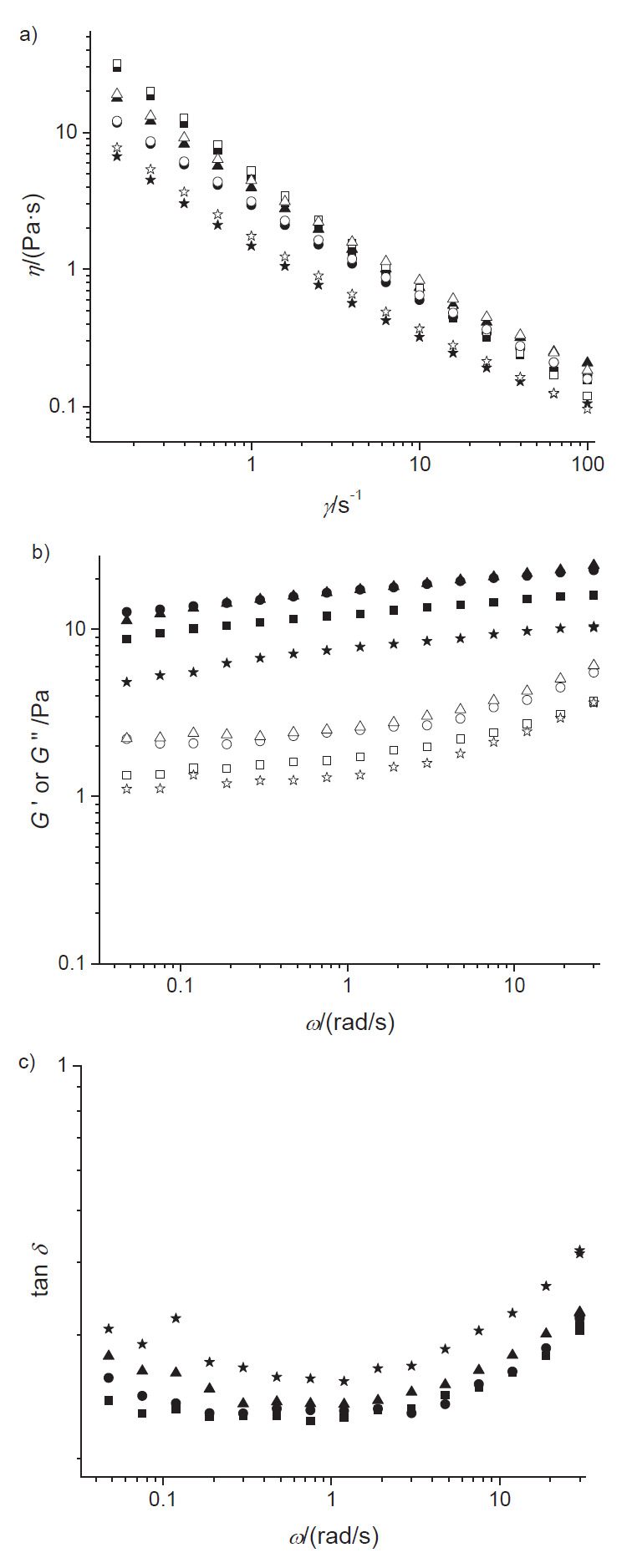
The curves obtained for: a) apparent viscosity for downward (closed symbols) and upward (open symbols) shear rate ramps, b) storage *G'* (closed symbols) and loss *G"* (open symbols) moduli as functions of frequency, and c) loss tangent, tan (*δ*) of the emulsions: O14S74P360 (*w*(buriti oil)=14%, *w*(SPI)=74% in wall material and *p*=360·10^5^ Pa) (squares), O26S56P360 (*w*(buriti oil)=26%, *w*(SPI)=56% in wall material and *p*=360·10^5^ Pa) (circles), O26S74P240 (*w*(buriti oil)=26%, *w*(SPI)=74% in wall material and *p*=240·10^5^ Pa) (triangles), and O26S74P360 (*w*(buriti oil)=26%, *w*(SPI)=74% in wall material and *p*=360·10^5^ Pa) (stars)

Fig. 1 shows the apparent viscosity curves, the storage and loss moduli as functions of frequency and loss tangent for the oil emulsions. Fig. 1a shows apparent viscosity curves corresponding to emulsions O14S74P360, O26S56P360, O26S74P240 and O26S74P360, which were chosen for presentation due to differences in their formulations (Table 1) and for possibility of comparing one variable at a time, while maintaining the other constant.

The emulsions showed similar shear-thinning behaviour, with their apparent viscosity decreasing with increasing shear rate. Comparing the emulsions O26S56P360 (circles) and O26S74P360 (stars) produced with the same oil mass fraction and homogenization pressure, slightly higher viscosity values were observed in the emulsion with the lower SPI mass fraction and, consequently, higher HMP mass fraction. On the other hand, comparing the emulsions O26S74P240 (triangles) and O26S74P360 (stars) that are of the same composition but homogenized at different pressures, it was noted that the higher homogenization pressure slightly reduced the apparent viscosity, probably because of the smaller oil droplets produced, which is in accordance with the discussion on the creaming index (see *Buriti oil emulsion stability*), as well as the results observed in previous works (*19*, *27*, *38*).

The Sisko model (Eq. 3) presented satisfactory fit to the apparent viscosity curves (Table 3), resulting in determination coefficients above 0.9 and low values of RMSE for both downward and upward shear rate ramps. The pseudoplastic behaviour could be explained by structure modification of the long chain molecules, such as those of SPI and HMP present in the continuous phase, as well as by modification of oil droplet aggregation due to the increase of velocity gradient, or shear rate (*2*). This behaviour has been commonly observed in concentrated emulsions (*6*, *19*, *27*, *38*). Infinite shear viscosity (*η*_∞_) provides information on how the system behaves when submitted to high shear conditions, such as spraying or extrusion (*34*). Under these conditions, the buriti oil emulsions or creamed phases would have reduced apparent viscosity but still higher than water viscosity.

**Table 3:** Consistency index (*K*), flow behaviour index (*n*), infinite shear viscosity () and determination coefficient (R^2^) for the Sisko model fitted to apparent viscosity curves and consistency index (*K’* and *K”*), flow behaviour index (*n’* and *n”*), determination coefficient (R*^2^*), and root mean squared error (RMSE) for the power law fitted to the viscoelasticity data of emulsions O14S74P360, O26S56P360, O26S74P240 and O26S74P360

Assay		Sisko viscosity model																	
Downward shear rate ramp									Upward shear rate ramp								
*K*/ (Pa·s^n^)		*n*		*η*∞/* (Pa·s)*		R^2^	RMSE		*K*/ (Pa·s^n^)		*n*		*η*∞/* (Pa·s)*		R^2^	RMSE	
O14S74P360		4.54		0.01		0.17		0.99	0.04		5.25		0.03		0.10		0.99	0.14	
O26S56P360		2.82		0.23		0.11		0.99	0.02		3.04		0.25		0.09		0.99	0.01	
O26S74P240		3.69		0.15		0.20		0.99	0.05		4.36		0.21		0.11		0.99	0.03	
O26S74P360		1.28		0.11		0.14		0.99	0.05		1.72		0.19		0.07		0.99	0.03	
Assay	Power law model fitted to frequency sweeps																			
	*K'*/ (Pa·s^n^*^’^*)		*n'*		R^2^		RMSE			*K"*/ (Pa·s^n”^)		*n"*		R^2^		RMSE			*n*_r,av_	
O14S74P360	12.10		0.09		0.99		0.20			1.83		0.18		0.93		0.20			0.13	
O26S56P360	16.76		0.09		0.99		0.13			2.61		0.19		0.87		0.41			0.14	
O26S74P240	16.75		0.11		0.99		0.30			2.84		0.19		0.89		0.42			0.15	
O26S74P360	7.41		0.11		0.98		0.27			1.49		0.23		0.89		0.27			0.17	

The apparent viscosity curves corresponding to the downward and upward shear rate ramps practically overlap, suggesting that the rheological behaviour of the emulsions was independent of the shearing time, which could be an interesting characteristic for industrial processing such as pumping and pipe flow.

#### Oscillatory shear emulsion behaviour

The storage (*G'*) and loss (*G"*) moduli as functions of frequency for the emulsions O14S74P360, O26S56P360, O26S74P240 and O26S74P360 are observable in Fig. 1b. In all the analyzed emulsions, *G'* showed values greater than *G"* in the entire studied frequency range, showing that the emulsions and creamed phases were elastic, a behavior characteristic of weak gel. Weak or physical gels are frequency dependent, with *G’*>*G”* but with no crossing-over, whereas true or elastic gels show constant *G’* along frequency sweeps (*33*, *43*). Unlike a true gel, which will break down when subjected to high shear (steady shear assays), a weak gel will flow under similar conditions (*44*, *45*).

The samples differed in storage and loss modulus magnitude, and the O26S74P360 emulsion (stars) had the lowest modulus. The O14S74P360 (squares) emulsion was the most unstable one, and despite this, its creamed phase showed similar viscoelastic results to O26S56P360 (circles) assay, which was a stable emulsion.

An appropriate way to analyze the mechanical spectra of biopolymer systems is based on fitting a power law model (Eqs. 4 and 5) to *G’* and *G” versus* frequency data. The fitting parameters obtained for the studied emulsions can be found in Table 3. It is possible to observe that the power law could be fitted to experimental data with high coefficients of determination (R^2^>0.87) and RMSE<0.43. For biopolymer systems that behave as true gels, the loss and storage moduli are congruent for several decades of frequency and this behaviour results in a constant value of the loss tangent, tan (*δ*), in which δ is the phase angle between stress (*σ*) and strain (*γ*). These relations can be expressed as follows (*46*, *47*):


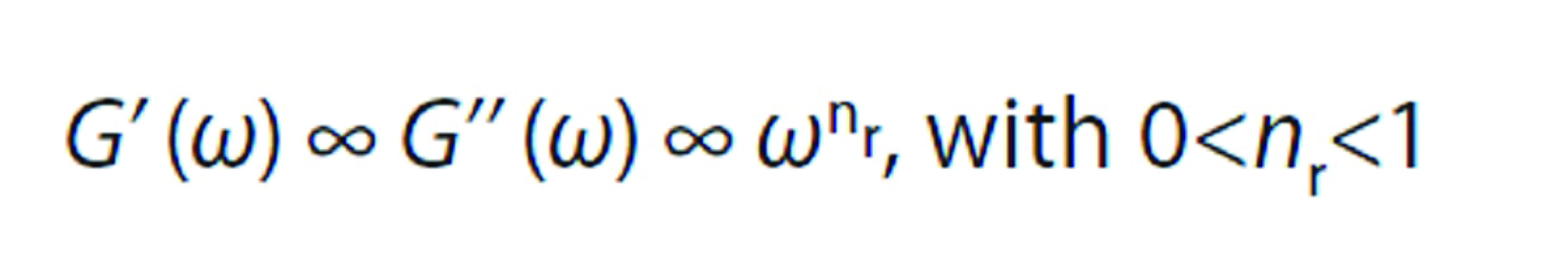


and


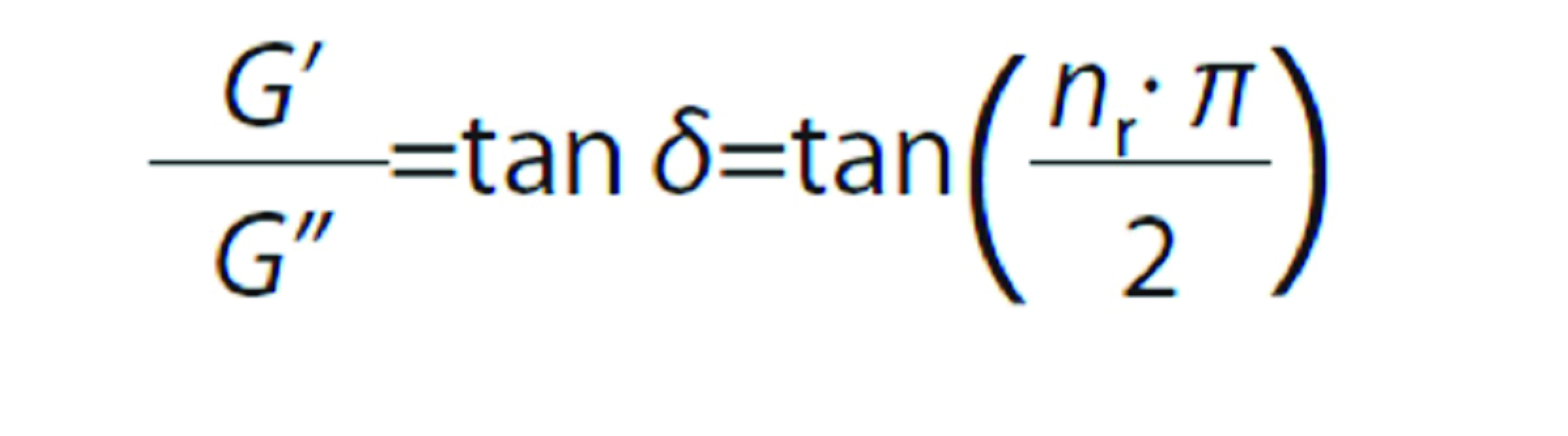


The parameter *n*_r_ is the relaxation exponent and Eq. 7 predicts that *G”*>*G’* for *n*_r_>0.5, *G’*>*G”* for *n*_r_<0.5, and *G”*=*G’* for *n*_r_=0.5. A gel with *n*_r_ approaching 1 is a purely viscous gel, whereas *n*_r_ approaching 0 suggests a purely elastic gel (*48*, *49*). Values of *n’* and *n”* can provide useful information regarding the viscoelastic nature of materials (*45*, *47*). The buriti oil emulsions or creamed phases showed *n’*<*n”*<0.24 (Table 3) and the relative magnitudes obtained for *n’* and *n”* are typical of weak gels, especially at low frequency. Values of tan (*δ*) were included in Fig. 1c.

### Buriti oil emulsion microscopic analyses

#### Optical microscopy of emulsions

**Fig. 2:**
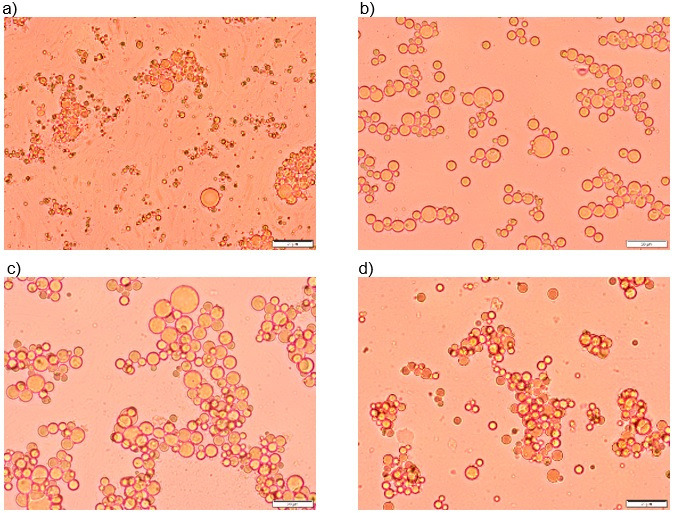
Images of emulsions: a) O14S74P360 (*w*(buriti oil)=14%, *w*(SPI)=74% in wall material and *p*=360·10^5^ Pa), b) O26S56P360 (*w*(buriti oil)=26%, *w*(SPI)=56% in wall material and *p*=360·10^5^ Pa), c) O26S74P240 (*w*(buriti oil)=26%, *w*(SPI)=74% in wall material and *p*=240·10^5^ Pa), and d) O26S74P360 (*w*(buriti oil)=26%, *w*(SPI)=74% in wall material and *p*=360·10^5^ Pa) obtained by optical microscopy with a 40× magnification objective (bars correspond to 20 μm)

Optical microscopy was used to observe the emulsion or creamed phase morphology and it was also possible to analyze and compare oil droplet arrangement among different assays. Fig. 2 shows the images collected with a 40× magnification of emulsions O14S74P360, O26S56P360, O26S74P240 and O26S74P360. It is possible to observe dispersed oil droplets in the continuous phase. In addition, these droplets were mostly arranged in clusters, suggesting that although the systems were negatively charged, these charges were not sufficient to maintain the oil droplets completely dispersed throughout the continuous phase, indicating that the emulsions were probably destabilized by the flocculation phenomena.

Emulsions O14S74P360 (Fig. 2a) and O26S74P360 (Fig. 2d) differ in oil content and larger oil droplets could be observed in assay O26S74P360 (Fig. 2d). This might confirm the model fitted in Table 2, in which the oil mass fraction had positive effect on the droplet size measured by optical microscopy. Emulsion O26S56P360 (Fig. 2b) was prepared with a lower SPI mass fraction in the wall material and therefore, a higher pectin mass fraction than O26S74P360 (Fig. 2d) emulsion. It was possible to observe that assay O26S56P360 had a higher pectin excess, which caused a higher negative charge and consequently lower flocculation. This agrees with the model in Table 2, in which the SPI content positively influenced the droplet size measured by LD. Emulsions O26S74P240 (Fig. 2c) and O26S74P360 (Fig. 2d) were produced using different homogenization pressures, and sample O26S74P360 had a smaller droplet size due to the higher homogenization pressure.

#### Confocal laser scanning microscopy of emulsions

**Fig. 3:**
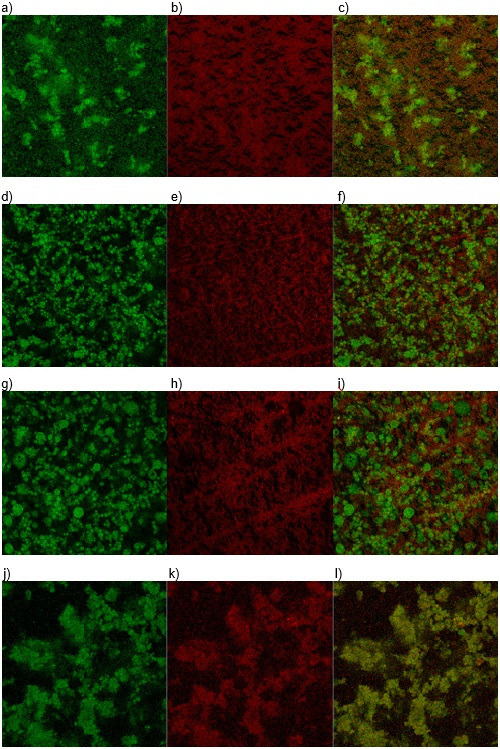
Images of emulsions: a-c) O14S74P360 (*w*(buriti oil)=14%, *w*(SPI)=74% in wall material and *p*=360·10^5^ Pa), d-f) O26S56P360 (*w*(buriti oil)=26%, *w*(SPI)=56% in wall material and *p*=360·10^5^ Pa), g-i) O26S74P240 (*w*(buriti oil)=26%, *w*(SPI)=74% in wall material and *p*=240·10^5^ Pa), and j-l) O26S74P360 (*w*(buriti oil)=26%, *w*(SPI)=74% in wall material and *p*=360·10^5^ Pa) obtained by confocal laser scanning microscopy with a 40× magnification. SPI can be seen in green (a, d, g, j), HMP in red (b, e, h, k) and their interaction can be observed as the orange regions in the overlaps (c, f, i, l)

The images corresponding to emulsions O14S74P360, O26S56P360, O26S74P240 and O26S74P360 (Fig. 3) were obtained in a confocal microscope with a 40× magnification. SPI is visible in green and HMP in red due to interaction with fluorescent dyes FITC and Rhodamine B, respectively.

It is observable that the SPI covered the oil droplets, which are seen as green spheres when observed individually (Fig. 3a, Fig. 3d, Fig. 3g and Fig. 3j). High-methoxyl pectin, in turn, interacted with protein and this interaction could be observed as the orange regions in the overlaps (Fig. 3c, Fig. 3f, Fig. 3i and Fig. 3l). In addition, HMP is also dispersed in the continuous phase appearing as a net when observed individually (Fig. 3b, Fig. 3e, Fig. 3h and Fig. 3k) and in red regions in overlapped images. The pectin excess in the samples seems to contribute to emulsion stability, preventing droplet movement, in addition to increasing the repulsion among them due to excess of negative charges.

Emulsion O26S56P360 (Figs. 3d-f) was stable for more than 7 days and a different microstructure from the others is noticeable, having smaller droplets that are homogeneously distributed in the continuous phase, with lower degree of flocculation. On the other hand, droplet clusters could be clearly visible in the images of assays O14S74P360 (Figs. 3a-c) and O26S74P360 (Figs. 3j-l).

#### Scanning electron microscopy of emulsions

**Fig. 4:**
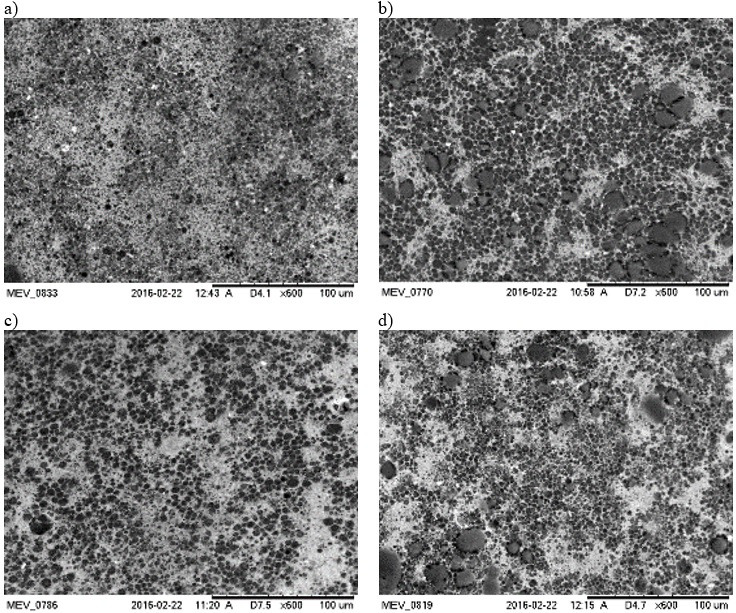
Images of emulsions: a) O14S74P360 (*w*(buriti oil)=14%, *w*(SPI)=74% in wall material and *p*=360·10^5^ Pa), b) O26S56P360 (*w*(buriti oil)=26%, *w*(SPI)=56% in wall material and *p*=360·10^5^ Pa), c) O26S74P240 (*w*(buriti oil)=26%, *w*(SPI)=74% in wall material and *p*=240·10^5^ Pa), and d) O26S74P360 (*w*(buriti oil)=26%, *w*(SPI)=74% in wall material and *p*=360·10^5^ Pa) by scanning electron microscopy at 600× magnification

The microstructure of emulsions or creamed phases was also analyzed by scanning electron microscopy. Fig. 4 shows images collected at 600× magnification of emulsions O14S74P360, O26S56P360, O26S74P240 and O26S74P360.

It is possible to observe a dense continuous phase, probably due to the excess of high-methoxyl pectin and buriti oil droplets embedded into this biopolymer matrix. Comparing the images, once more a difference in morphology is observable comparing different formulations. The droplets of O14S74P360 emulsion (Fig. 4a), for example, are small and more flocculated, with regions containing only continuous phase (lighter regions). Droplets of different sizes could be noted comparing images of O26S56P360 (Fig. 4b) and O26S74P360 (Fig. 4d) emulsions.

### Experimental validation of the models obtained for emulsion production

In order to produce a stable emulsion with high carotenoid and tocopherol contents, which corresponds to a higher buriti oil content, with reduced size droplets, low electrical conductivity, and high electrical charge, the optimal values for the independent variables were fixed at 28% buriti oil, 55% SPI, and homogenization pressure of 380·10^5^ Pa.

The observed results, as well as those predicted by the regression models (Table 2) for the validation assay are shown in Table 4, as well as the fitting and relative errors. The obtained results were consistent with those expected and, due to proximity of the experimental and predicted results, the relative errors were low, suggesting that the experimental design was adequate to obtain predictive models for the production of buriti oil emulsions.

**Table 4:** Experimental results and values predicted by the regression models and errors for the rotatable central composite design (RCCD) validation assay

Analysis	Experimental result	Predicted result	Fitting error	Relative error/%
CI/%	0.0±0.0	-	-	-
*d*(droplet OM)/μm	4.6±1.1	5.01	0.43	8.58
*d*(droplet LD)/μm	38.2±0.3	44.47	6.29	14.14
*σ*/(mS/cm)	2.23±0.03	2.34	0.11	4.70
*ζ*/mV	-17.7±0.7	-20.11	-2.46	12.23

-=The model predicts that there would be no creaming index (CI). OM=optical microscopy, LD=lase diffraction

The images obtained by optical microscopy, SEM and CLSM allowed to observe that the optimized buriti oil emulsion morphology is small and less flocculated.

The pseudoplastic behaviour of this system was confirmed by the good fitting of the Sisko viscosity model. The mechanical spectra revealed that the sample was located in the viscoelastic plateau region and its microstructure revealed weak gel characteristics.

The results obtained in the present work may be compared to similar investigations reported in literature. Goyal *et al*. (*50*) developed a stable flaxseed oil emulsion with 12.5% oil, 7.5% WPC-80 and 10% lactose (filler), homogenized at 207·10^5^ Pa, which was suggested to be potentially applied as ω-3-fatty acid delivery system in functional foods such as pastry, ice-creams, curd, milk, yogurt, cakes, *etc*. Djordjevic *et al*. (*51*) studied physical stability of whey protein‐stabilized O/W emulsions at pH=3 using corn oil, and concluded that the emulsions could be used to incorporate functional lipids that are sensitive to oxidation. In addition, Nikovska (*52*) studied the oxidative stability of walnut oil and O/W emulsions containing walnut oil stabilized by SPI and whey protein isolate, observing that the emulsions were more stable than pure walnut oil. Based on these observations, the next steps of the present study should focus on the investigation of chemical composition and chemical stability of carotenes and other relevant constituents of buriti oil emulsions produced applying the optimized formulation developed in this work.

## CONCLUSIONS

The rotatable central composite design (RCCD) was suitable for investigating the formation of buriti oil emulsions stabilized by soy protein isolate (SPI) and high-methoxyl pectin (HMP) electrostatic interactions. Thus we obtained an optimized emulsion produced with 28% buriti oil, 55% SPI in the continuous phase, and homogenized at 380·10^5^ Pa, which was stable for at least 7 days, having reduced droplet size (4.58 µm), low electrical conductivity (2.23 mS/cm), and high modulus of negative charges (-17.65). This emulsion behaved typically for a weak gel and an excess of pectin in the continuous phase was observed to contribute to the emulsion stability. Emulsions containing the carotenoid-rich buriti oil stabilized by SPI/HMP could be valuable structured systems applied for fat substitution, energy reduction, and carotenoid enrichment in foods such as dairy and bakery products, ice cream, salad sauces, and vegetable-based cream.
